# Segmenting Based on UNETR Network and 3D Reconstruction of Interventricular Septal-Free Wall Structure

**DOI:** 10.1155/abb/2610376

**Published:** 2025-08-14

**Authors:** Yupeng Han, Chao Han, ShengJun Ta, Rui Hu, JiaQi Yin, Xin'an Li, Liwen Liu

**Affiliations:** Department of Ultrasound, Hypertrophic Cardiomyopathy Center, Xijing Hospital, Fourth Military Medical University, Xi'an 710032, Shaanxi, China

**Keywords:** hypertrophic cardiomyopathy, neural network–based, PIMSRA, three-dimensional reconstruction, UNETR

## Abstract

**Background:** Percutaneous intramuscular septal radiofrequency ablation (PIMSRA) provi des an innovative alternative to septal myectomy (SM) and alcohol septal ablation (ASA). The precise segmentation of the interventricular septal is of paramount importance for the successful execution of the PIMSRA procedure. The study is based on the feasibility of applying the neural network-based method of UNETR model to the automatic segmentation of ventricular septal free wall structure in cardiac CT images of patients with HOCM and the three-dimensional reconstruction method based on visualization toolkit (VTK).

**Materials and Methods:** The MedSAM-2, UNET and UNETR models were used to automatically segment the interventricular septal wall structure from 700 cardiac CT images of 23 patients. The image annotation tool was Labelme software, and the ratio of training set, test set, as well as validation set was 6:2:2. The higher Dice coefficient of the segmentation model was chosen to address the images, and the segmentation results were uesd to reconstruct in 3D with the metod of moving cubes.

**Results:** Via training the UNETR segmentation model, when the parameter of batch and epoch were 8 and 32, respectively, the Dice coefficient of the interventricular septal-free wall structure test set is 0.89, which is higher than the Dice coefficient of 0.81 of the UNET model and 0.83 of the MedSAM-2 model. The model of UNETR was chosen to achieve the better segmentation results, and VTK three-dimensional reconstruction was performed based on the better segmentation results which is more closer to the real structure of heart.

**Conclusion:** The results show that the segmentation method is feasible, and the three-dimensional reconstruction of the interventricular septal free wall structure by VTK based on the segmentation results is also feasible.

## 1. Introduction

Hypertrophic cardiomyopathy (HCM) is an autosomal dominant myocardial disease characterized by asymmetric myocardial hypertrophy, with a incidence rate of 1:500–1:200 [1, 2]. For HCM patients who are unresponsive to drug therapy, Septal reduction therapy (SRT) is a reasonable choice, including septal myectomy (SM) and alcohol septal ablation (ASA). Percutaneous intramuscular septal radiofrequency ablation (PIMSRA) provides an innovative alternative to SM and ASA [3–5]. The excellent clinical outcome and relatively low incidence of complications of PIMSRA in treating HCM patients have received widespread attention from peers. However, the precise segmentation of the septal is of paramount importance for the successful execution of the PIMSRA procedure. The current method of septal segmentation relies on the integration of two-dimensional ultrasound sections captured from various angles, which are subsequently mentally reconstructed into a three-dimensional spatial configuration by the surgeon. This approach necessitates a high level of expertise and cognitive ability from the surgeon. Hence, there exists an imperative requirement for a 3D reconstruction methodology specifically tailored for septal 3D reconstruction. This technique is indispensable for preoperative planning and intraoperative guidance, aiming to refine and optimize surgical strategies.

Based on CT or MRI images, the techniques of segmentation and 3D reconstruction have been extensively utilized within the medical domain. Nevertheless, their application in the realm of three-dimensional reconstruction of the interventricular septal remains unprecedented.

Neural network-based methods have been widely applied in the field of medical image analysis [6–10], and target segmentation has important value in three-dimensional visualization, surgical planning, and quantitative image analysis. UNETR is a segmentation model tailored for three-dimensional segmentation and directly utilizes CT volume data. The UNETR model utilizes the powerful self attention mechanism of transformer to effectively capture global multiscale information, while following the design of U-shaped network encoder and decoder to improve segmentation performance. Establishing a 3D model based on the segmentation results of the target area is beneficial for doctors to conduct preoperative planning, and develop reasonable and personalized surgical strategies. Therefore, given the urgent clinical needs and technological limitations, researchers are encouraged to continue developing methods for segmentation, and 3D reconstruction of the interventricular septal-free wall structure in HCM, which will be a challenging task.

## 2. Materials and Methods

### 2.1. Related Research

Due to the thickening of the interventricular septal in HCM patients, which leads to changes in cardiac morphology, and the fact that there is no visual difference between the interventricular septal and adjacent tissues, these characteristics pose big challenges to the intelligent addressing of CT images of HCM patients. Therefore, automatic segmentation of the interventricular septal-free wall structure is a very challenging task. For the intelligent processing of cardiac anatomy, there have been early researches on whole heart structure detection [11–15]. With the improvement of deep learning algorithms and models, tasks have gradually shifted from region object detection to object segmentation research [16], from single anatomical landmarks to multiple anatomical landmarks, and from two-dimensional segmentation to three-dimensional segmentation [17]. In previous whole heart segmentation tasks, in addition to training on a single two-dimensional image, 2D images adjacent to the heart were also used for training to increase spatial information. The main selection of segmentation models is based on UNET networks and their improved networks. The training of two-dimensional networks requires less computer hardware than three-dimensional networks, but two-dimensional images will lose a lot of effective spatial information, resulting in insufficient sensitivity and accuracy in detection. Three-dimensional networks generally have a better segmentation accuracy, but are often limited by hardware and cannot involve larger and broader networks.

Compared to whole heart segmentation, the interventricular septal-free wall structure has a wider diameter range and is susceptible to interference from other tissue structures, lesions, noise, and other factors. In response to these existing problems, this article is committed to achieving automatic segmentation and 3D reconstruction of the interventricular septal-free wall structure in HCM cardiac CT images based on the latest theoretical foundations.

The purpose of this research is to evaluate the segmentation performance of UNETR segmentation network on interventricular septal-free wall structure in cardiac CT images of HCM patients, compare it with UNET segmentation network, and perform 3D reconstruction based on UNETR segmentation results. We use CT images of HCM patients' hearts for training, testing, and validation, providing a model foundation for future implementation of safe and effective personalized PIMSRA diagnosis and treatment plans.

### 2.2. Network Model

Since the emergence of UNET, CNN based networks have been widely used for various 2D and 3D medical image segmentation tasks. In this study, UNET and UNETR networks were used to automatically segment the interventricular septal-free wall structure in cardiac CT images of HCM patients.

**Figure 1:**
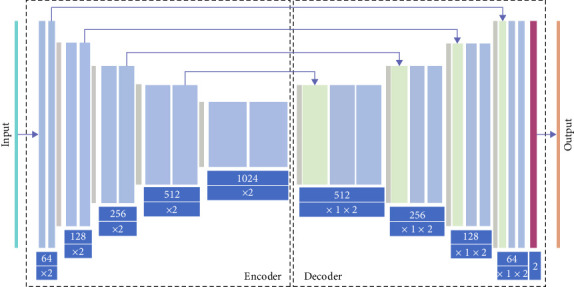
UNET segmentation network.

UNET [18] segmentation network (Figure 1) directly uses a full volume image composed of a series of two- dimensional slices, UNET. The use of convolutions of different sizes for multiscan and multipath fusion to capture downsampling features of images provides groundbreaking research on multilevel CT image segmentation and reduces the problems of spatial background and low resolution conditions. UNET models have made significant breakthroughs in segmentation tasks, but their limitations lie in their lower performance in studying global context and long-range spatial dependencies, resulting in poor image segmentation performance. The role of visual converters in computer vision missions is receiving more attention. Through conducting large-scale pretraining and fine-tuning on pure transformers, advanced performance has been demonstrated on image classification datasets.

**Figure 2:**
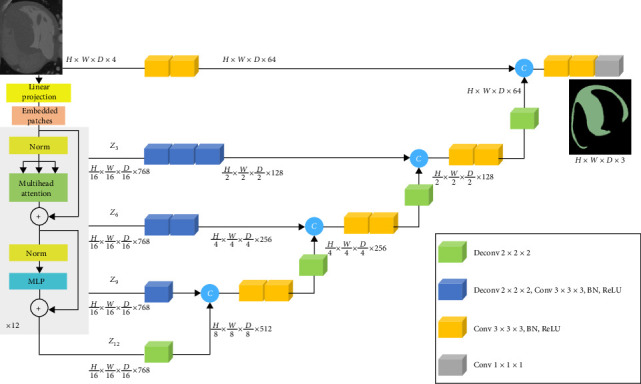
UNETR segmentation network.

The UNETR segmentation network [19] (Figure 2) continues to advantage the classic frame called encoder to decoder structure. However, this frame contains transformer as the encoder to study the input sequence representation and effectively capture global multiscale information. The transformer encoder is directly linked to the decoder via skip connections of different resolutions to calculate the final semantic segmentation output. This is the first proposed transformer segmentation model specifically designed for three-dimensional image segmentation, which can redefine the mission of three-dimensional medical image segmentation as a sequence to sequence prediction problem. Transformers are commonly used as backbone encoders in computer vision due to their powerful ability to model long-term dependencies and capture global context. Generally speaking, unlike convolution methods, transformers use a stack of transformer modules consisting of multi head self attention (MSA) and multilayer perceptron (MLP) in sub layers to encode images into one-dimensional image embedding sequences.

**Figure 3:**
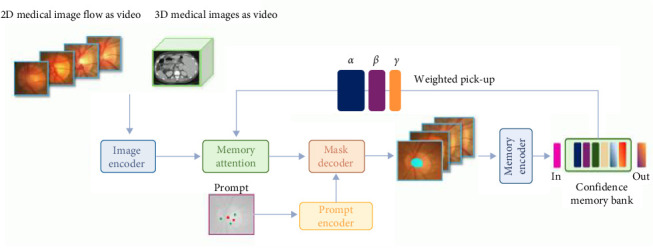
MedSAM-2 segmentation network.

In 2024, a technical team from the University of Oxford developed the Medical SAM-2 (MedSAM-2) segmentation network [20] (Figure 3) medical image segmentation model, which is designed based on the SAM-2 framework. It can provide real-time and suggestive object segmentation for static images and dynamic video content, and integrate image and video segmentation functions into one system. The MedSAM-2 model not only performs excellently in 3D medical image segmentation tasks, but also unlocks a new ability for single prompt segmentation. Users only need to provide a prompt for a new specific object, and the segmentation of similar objects in subsequent images can be automatically completed by the model without further input.

### 2.3. Loss Function

In this study, the loss function stands a combination of Dice and cross entropy loss. The index of *I* can be calculated in the manner of voxels, that is,

  $$\begin{array}{c} L=1-\frac{2}{J}\sum_{j=1}^{J}\frac{\sum_{i=1}^{I}G_{i,j}Y_{i,j}}{\sum_{i=1}^{I}G_{i,j}^{2}+\sum_{i=1}^{I}Y_{i,j}^{2}}-\frac{1}{I}\sum_{i=1}^{I}\sum_{j=1}^{J}G_{i,j}\log Y_{i,j}, \end{array}$$

where *I* stands the number of voxels; *J* stands the number of classes; *Y*_*i*,*j*_ and *G*_*i*,*j*_ represents the predicted and true values of class *J* at voxel *I*, respectively.

### 2.4. Datasets and Preprocessing

**Figure 4:**
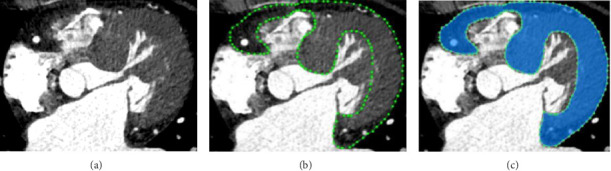
Professional software was used to delineate the structure of the interventricular septal-free wall. (a) HCM CT data; (b) Labelme software mark the target structure; (c) the structure of the interventricular septal-free wall.

The cardiac CT data for this study was sourced from the open-source dataset MM-WHS and a total of 700 CT images from 23 patients at the First Affiliated Hospital of the Air Force Medical University. Manual segmentation was performed by professional labelers who were trained by doctors with more than 15 years of experience specializing in HCM. To ensure consistency, multiple experts reviewed each annotation, and any discrepancies were resolved collaboratively. Additionally, the annotations were cross-checked for accuracy, and the final dataset was reviewed to ensure that the segmentation labels were reliable for training. The data modality was CT scanning, and the data recording format was. jpg screenshots. The annotation tool was labelme software, and the training, testing, as well as validation sets were divided in a ratio of 6:2:2. The results were shown in Figure 4.

### 2.5. Experimental Environment and Hyperparameters

Experimental environment: CPU: Intel Xeon Platinum 8163 (Skylake) @ 2.5GHz, memory: 64GB, video card: Tesla V100. In this study, UNETR and UNET were used, without pretraining weight loading, and only original CT images were used. It is read as a 2D grayscale map and weighted sampling is carried out subject to the number of classes when loading the CT data to make the proportion of different samples in each batch as balanced as possible. Setting the batch size to 8 and epoch to 32, random horizontal flip, rotation, scaling, Gaussian blur, and other methods were used to enhance the data. In addition, images size scaled to (512,512).

### 2.6. Evaluation Indicators

In this research, Dice coefficient is trated as an assessment index, which is a usually used assessment index in CT image segmentation. This measure is basically the similarity (overlap ratio) between the segmentation result and the corresponding picture. Dice ranges from 0 to 1, where 1 stands that the segmentation result totally overlaps the real segmentation outcome. The calculation formula is defined as:

  $$\begin{array}{c} \text{Dice }\left(A,B\right)=\frac{2\times \left|A\cap B\right|}{A+B}, \end{array}$$

Where *A* stands the predicted segmentation outcome and *B* shows the real segmentation outcome.

## 3. Results

### 3.1. Results of Segmentation

All images contained in 23 patients were used for training and testing of the model, and the experimental results were shown in Table 1 (the scores were Dice indicators). Ablation experiments show that both facets of random geometric enhancement and random Gaussian fuzzy data enhancement strategies can steadily upgrade the performance of the segmentation model, indicating that the data is more sensitive to such enhancement.

**Figure 5:**
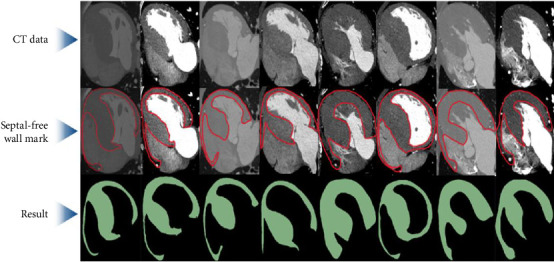
Segmentation of interventricular septal-free wall.

As shown in Figure 5, after expert annotation of the interventricular septal-free wall structure, the cardiac CT image is input into the UNETR network for image segmentation, and the segmented interventricular septal-free wall structure is finally obtained, which is used for the next step of 3D reconstruction of the interventricular septal free wall.

### 3.2. 3D-Reconstruction

The results obtained through UNETR segmentation based on two-dimensional CT images require three-dimensional reconstruction to clearly understand the internal structure of the patient's heart. This study used the marching cubes [21]method for three-dimensional reconstruction of interventricular septal-free walls, which has the following advantages: (a) hardware acceleration can be used during drawing; (b) less memory usage; (c) changing the perspective, lighting, and so on, only requires redrawing, there is no need to rebuild again; (d) can compress storage and transmission; (e) draw in the order of graphic objects, and do not draw what is not visible; (f) clear spatial location and high drawing efficiency.

**Figure 6:**
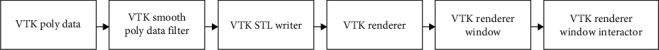
Drawing of pipeline model based on marching cube algorithm for the interventricular septal-free wall structural surface.

The segmented interventricular septal-free wall two-dimensional section must be reconstructed in three- dimensions to clearly understand the internal structure of the heart. In this study, the moving cube method was used for reconstruction, and the two-dimensional section source data was read sequentially; forming preliminary three-dimensional structures through visualization toolkit (VTK) marching cubes; VTK filter performs denoising on three-dimensional structures; finally, display the 3D data in the VTK renderer window interaction box. The result was shown in Figure 6.

**Figure 7:**
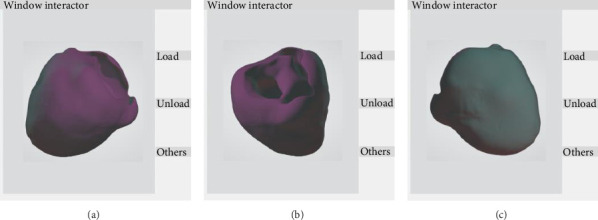
Interactive window based on VTK 3D reconstruction. (a) 3-D structure front view; (b) top view of three-dimensional structure; (c) side view of 3D structure.

VTK adopts the pipeline mechanism [21] and provides a series of interfaces for Python based on the VTK library. By writing relevant programs, it realizes the complete function of segmentation results from reading to 3D visualization, and then exporting binary.stereolithography (STL) format that can be used for grid division. The coronal, sagittal, and transverse planes of the interventricular septal-free wall structure were realistically displayed, and a three-dimensional solid shape was reconstructed, as well as corresponding two-dimensional planes cut at any angle. The result were shown in Figure 7.

**Figure 8:**
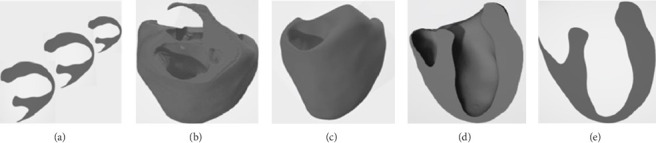
(a) Multilayer cross section of the chamber wall; (b) stacked three-dimensional wall structure; (c) three-dimensional wall structure after denoising; (d) cutting the three-dimensional the interventricular septal-free wall structure along the longitudinal axis; (e) 2D section extraction.

In Figure 8, by using the UNETR model for automated and batch segmentation of interventricular septal-free wall structures, as well as VTK 3D reconstruction based on segmentation results, this research method can obtain two-dimensional and three-dimensional information of interventricular septal-free wall for different types of hypertrophy. This will be beneficial for doctors to develop safe, effective, and personalized treatment plans.

## 4. Discussion

In previous researches, automatic segmentation of CT images was mainly based on UNET models and their improved networks, such as enrolling attention mechanisms, dense connection mechanisms, dilated convolutions, and so on. Those improvements can hoist the efficiency of the segmentation model, however, there are still certain disadvantages in the segmentation of small landmarks in CT images of HCM patients.

Since its proposal in 2021, UNETR has revealed good performance in the segmentation of brain tumors and spleens, characterized by the following aspects: (1) UNETR is specifically used for three-dimensional segmentation and utilizes CT data directly; (2) UNETR uses transformers as the principal encoder for segmented networks and directly links them to decoders via skip connections, rather than operating them as attention layers within segmented networks; (3) UNETR fails to rely on the backbone CNN to produce input sequences and uses tagged patches directly. UNETR reconstructs the three-dimensional segmentation mission into a one-dimensional sequence to sequence prediction matter, and utilizes the transformer structure as an encoder to study contextual information from embedded input patches. The representation extracted from the transformer encoder is merged with the decoder at multiple resolutions through skip connections to predict the segment output.

In the segmentation network, if the batch size is inadequated, then the algorithm would fail to converge. With the number of batches increasing, the algorithm will process the same amount of data becomes faster and efficient. With the number of batches increasing, more epochs are needed to obtain the same accuracy. Owing to the contradiction between the batch and epoch, the batch is added to a certain time to reach the optimal time. Due to the final convergence accuracy reaching different local optima, the batch size is increased to a certain point to obtain the best final convergence accuracy.

Accurate morphological evaluation of the interventricular septal in HCM patients will help doctors choose effective ablation needle placement schemes and safe puncture path planning before surgery. It should be noted that the types of ventricular septal hypertrophy in HCM patients are diverse and structurally complex, even difficult to recognize with the naked eye, requiring extensive training in this structure. Only professional doctors can correctly evaluate its possibility, which is a disadvantage that cannot be overlooked. Due to this fact, the segmentation of ventricular septal-free wall structure in HCM patients is worthwhile.

This study still has certain limitations. Occasional artifacts may exist in cardiac CT images of HCM patients, leading to poor generalization ability of segmentation models. More experienced radiologists need to carefully confirm the target area annotation in the images. In our study, we employed several strategies to enhance the generalization of the model, such as extensive data augmentation, including random horizontal flips, rotations, scaling, and Gaussian blur. These techniques were applied to increase the diversity of the training data. However, we acknowledge that further improvements in generalization could be achieved by including a more varied dataset, which could involve a larger number of patients, diverse pathological conditions, and different imaging protocols. Furthermore, to boost the model's robustness, we are exploring the possibility of incorporating advanced techniques like domain adaptation and transfer learning in future studies.

In future research, we will emphasize on increasing the sample size and promoting quality of the training set for multiple types of HCM patients.

## 5. Conclusion

This study used the UNETR segmentation model to acquire automatic and robust segmentation of interventricular septal-free wall structures in cardiac CT images of HCM patients, and obtained the better segmentation performance than the UNET and MedSAM-2 model. Therefore, the UNETR segmentation model is appropriate for segmenting the interventricular septal-free wall structure in cardiac CT images of HCM patients. Meanwhile, VTK 3D reconstruction technology based on the moving cube method for interventricular septal-free wall structure is also feasible. Doctors use three-dimensional models to make multidimensional judgments on the ablation target area and develop safe, effective, and personalized preoperative treatment strategies.

## Figures and Tables

**Table 1:** Dice coefficients of the three models on the four facets.

Model	MedSAM-2	UNET	UNETR
Basic model	0.82	0.78	0.72
Basic model + random horizontal flip	0.82	0.81	0.77
Basic model + random horizontal flip + immediate rotation zoom	0.83	0.83	0.81
Basic model + random horizontal flip + immediate rotation zoom + random Gaussian fuzzy	0.83	0.81	0.89

## Data Availability

The data that support the findings of this study are available upon request from the corresponding author. The data are not publicly available due to privacy or ethical restrictions.

## References

[1] Marian A. J., Braunwald E. (2017). Hypertrophic Cardiomyopathy: Genetics, Pathogenesis, Clinical Manifestations, Diagnosis, and Therapy. *Circulation Research*.

[2] Semsarian C., Ingles J., Maron M. S., Maron B. J. (2015). New Perspectives on the Prevalence of Hypertrophic Cardiomyopathy. *Journal of the American College of Cardiology*.

[3] Liu L., Li J., Zuo L. (2018). Percutaneous Intramyocardial Septal Radiofrequency Ablation for Hypertrophic Obstructive Cardiomyopathy. *Journal of the American College of Cardiology*.

[4] Zhou M., Ta S., Hahn R. T. (2022). Percutaneous Intramyocardial Septal Radiofrequency Ablation in Patients With Drug-Refractory Hypertrophic Obstructive Cardiomyopathy. *JAMA Cardiology*.

[5] Liu L., Liu B., Li J., Zhang Y. (2018). Percutaneous Intramyocardial Septal Radiofrequency Ablation of Hypertrophic Obstructive Cardiomyopathy: A Novel Minimally Invasive Treatment for Reduction of Outflow Tract Obstruction. *EuroIntervention*.

[6] Lin L., Dou Q., Jin Y.-M. (2019). Deep Learning for Automated Contouring of Primary Tumor Volumes by MRI for Nasopharyngeal Carcinoma. *Radiology*.

[7] Li Z., Zhou L., Tan S., Tang A. (2023). Application of UNETR for Automatic Cochlear Segmentation in Temporal Bone CTs. *Auris Nasus Larynx*.

[8] Bizopoulos P., Koutsouris D. (2019). Deep Learning in Cardiology. *IEEE Reviews in Biomedical Engineering*.

[9] Gillot M., Baquero B., Le C. (2022). Automatic Multi-Anatomical Skull Structure Segmentation of Cone-Beam Computed Tomography Scans Using 3D UNETR. *PLoS ONE*.

[10] Kim H. S., Kim H., Kim S. (2024). Precise Individual Muscle Segmentation in Whole Thigh CT Scans for Sarcopenia Assessment Using U-Net Transformer. *Scientific Reports*.

[11] Zhuang X. (2013). Challenges and Methodologies of Fully Automatic Whole Heart Segmentation: A Review. *Journal of Healthcare Engineering*.

[12] Zhuang X., Song J., Zhan S. A Registration and Atlas Propagation Based Framework for Automatic Whole Heart Segmentation of CT Volumes.

[13] Payer C., Štern D., Bischof H., Urechler M. Multi-Label Whole Heart Segmentation Using CNNs and Anatomical Label Configurations.

[14] Yang X., Bian C., Yu L., Ni D., Heng P. A. Hybrid Loss Guided Convolutional Networks for Whole Heart Parsing.

[15] Dou Q., Ouyang C., Chen C., Glocker B., Zhuang X., Heng P. A. (2018). PnP-AdaNet: Plug-and-Play Adversarial Domain Adaptation Network With a Benchmark at Cross-Modality Cardiac Segmentation.

[16] Zhou X., Wang S., Chen H. (2012). Automatic Localization of Solid Organs on 3D CT Images by a Collaborative Majority Voting Decision Based on Ensemble Learning. *Computerized Medical Imaging and Graphics*.

[17] Habijan M., Galic I., Levwntic H., Romic K. (2021). Whole Heart Segmentation Using 3D FM-Pre-ResNet Encoder–Decoder Based Architecture With Variational Autoencoder Regularization. *Applied Sciences*.

[18] Ronneberger O., Fischer P., Brox T. U-Net: Convolutional Networks for Biomedical Image Segmentation.

[19] Hatamizadeh A., Tang Y. C., Nath V. UNETR: Transformers for 3D Medical Image Segmentation.

[20] Zhu J., Qi Y., Wu J. (2024). Medical Sam 2: Segment Medical Images as Video via Segment Anything Model.

[21] Wang Y. A., Wan W. G., Wang R. An Improved Interpolation Algorithm Using Nearest Neighbor From VTK.

